# Bullous Ischemic Necrosis of the Penile Shaft With Periurethral Sparing: A Novel Cutaneous Manifestation of Trazodone-Induced Priapism

**DOI:** 10.7759/cureus.83571

**Published:** 2025-05-06

**Authors:** Vincent Gao, Benjamin W Casterline, Kari Martin

**Affiliations:** 1 Dermatology, University of Missouri School of Medicine, Columbia, USA; 2 Dermatology, University of Missouri, Columbia, USA

**Keywords:** bullous ischemic necrosis, ischemic priapism, medical treatment of ischemic priapism, priapism refractory to treatment, recurrent priapism, trazodone, trazodone-induced priapism

## Abstract

Priapism is associated with hematologic disorders such as sickle cell disease and a rare adverse effect of psychotropic medications, including trazodone. While full-thickness penile gangrene is a rare but recognized complication of prolonged ischemic priapism, other cutaneous complications have not been described. We present an unprecedented case of a 40-year-old male who developed vesiculobullous necrosis with distinctive periurethral sparing following trazodone-induced priapism. The patient presented with a 15-hour priapism requiring surgical intervention with a modified proximal shunt, followed by the development of tense bullae on the penile shaft 48 hours after the procedure. We propose that this atypical morphology and distribution reflects distinctive features of the vascular anatomy of the penis, where the glans and periurethral tissues receive redundant blood supply from the terminal branches of the internal pudendal artery, while the shaft skin depends on superficial vessels vulnerable to compression during elevated intracavernosal pressures. The 48-hour delay between intervention and vesiculation is consistent with ischemia-reperfusion injury, a mechanism involving oxidative damage and inflammation triggered by the restoration of blood flow to ischemic tissues. This case represents a novel cutaneous manifestation of drug-induced priapism that clinicians should distinguish from other vesiculobullous disorders. Recognition of the characteristic distribution may permit clinical diagnosis without the need for biopsy, allowing for appropriate conservative management.

## Introduction

Priapism is a condition characterized by prolonged erection of the penis without appropriate sexual stimulation. While commonly associated with sickle cell disease, priapism can also be a rare side effect of several psychotropic medications, including both typical and atypical antipsychotics, as well as trazodone [1,2]. While the precise mechanism remains unclear, it is widely believed that priapism occurs due to the alpha-adrenergic blocking effects of these drugs [2]. Ischemic priapism is a medical emergency that necessitates immediate intervention to prevent penile damage and permanent erectile dysfunction. Early treatment is crucial for preserving erectile function. Without such interventions, necrosis of penile corporal tissue and subsequent fibrosis can occur, leading to irreversible erectile dysfunction and even leading to penile gangrene.

We report a unique case of a 40-year-old man who developed trazodone-induced ischemic priapism and subsequently presented with sharply demarcated vesiculation and tense bullae across the penile shaft, sparing the periurethral region. Initially suspected to be contact dermatitis, the pattern and healing course were more consistent with localized bullous ischemic necrosis. This regional distribution likely reflects underlying penile vascular anatomy, where the glans and periurethral tissues benefit from more redundant arterial supply compared to the shaft. To our knowledge, this represents the first reported case of such a cutaneous manifestation in the setting of drug-induced priapism.

## Case presentation

A 40-year-old male with a history of substance abuse presented to the emergency department for evaluation of priapism. The patient denied any sildenafil use, straddle injury, or a history of sickle cell disease. He reported taking his regularly prescribed dose of trazodone, which he had been using for the past eight months. He complained of severe pain with no discoloration of the penis. No tenderness or abnormality was noted on examination of the scrotum or testicles. Initial management included bedside aspiration and irrigation of the corpora cavernosa, followed by intracavernosal phenylephrine injection, which resulted in temporary detumescence. However, the recurrence of the erection prompted intervention with corporal decompression and modified proximal shunting.

**Figure 1:**
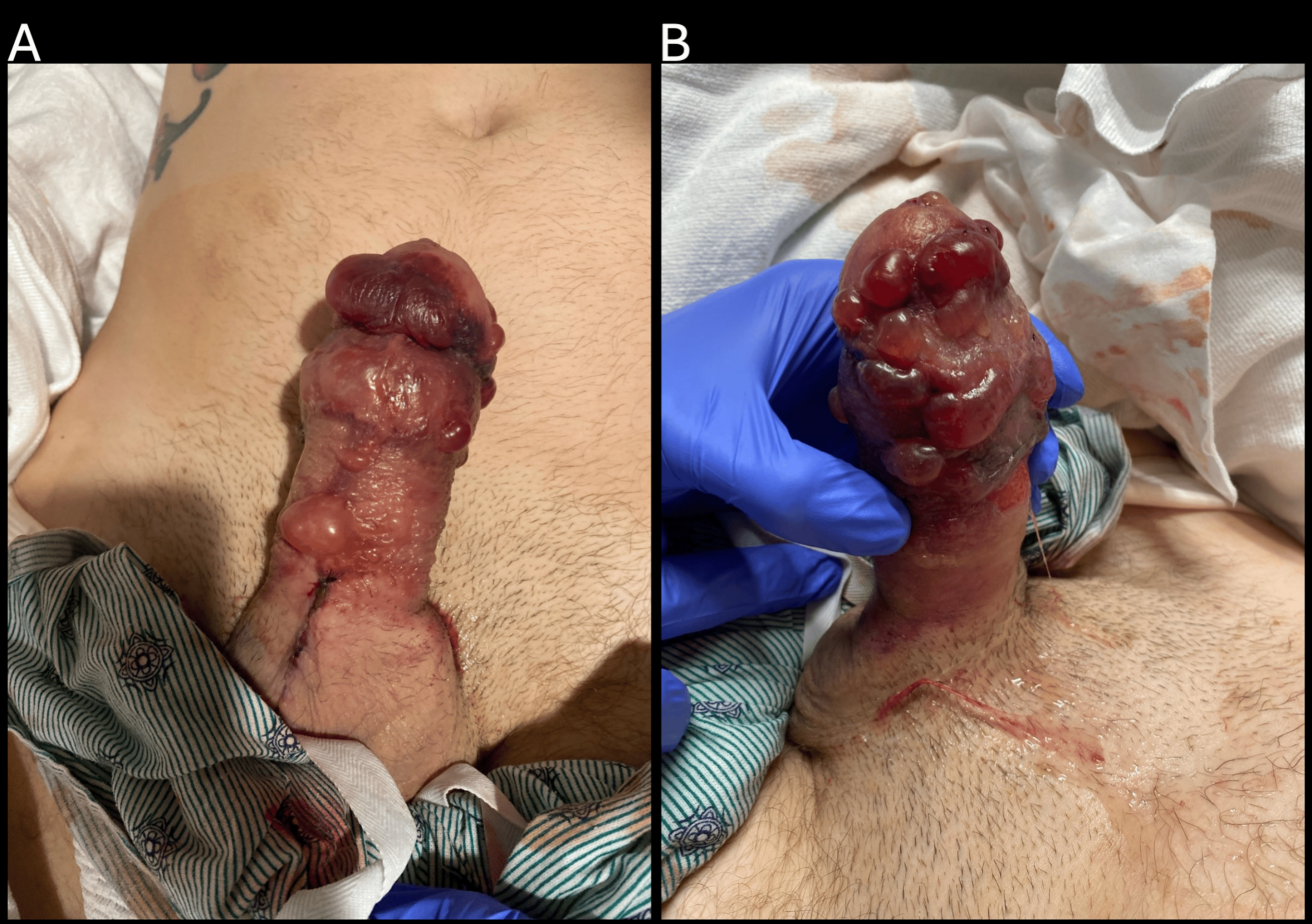
Diffuse vesiculation progressing into tense bullae on the penile shaft and glans, containing serous to serosanguineous fluid. The scrotum and base of the penis, including the proximal aspect of the surgical incision, are spared.

Two days postoperatively, the patient reported waxing and waning pain and return of erection. Removal of the bandaging revealed increased penile rigidity and bruising and blistering of the skin (Figure 1). Dermatology was consulted and suspected vesiculobullous allergic contact dermatitis, linear IgA bullous dermatosis to vancomycin, fixed drug eruption, or edema bullae. Allergic contact dermatitis was favored due to the morphology and sharp demarcation to the shaft of the penis, and the affected area was treated with topical clobetasol 0.05% ointment twice daily.

**Figure 2:**
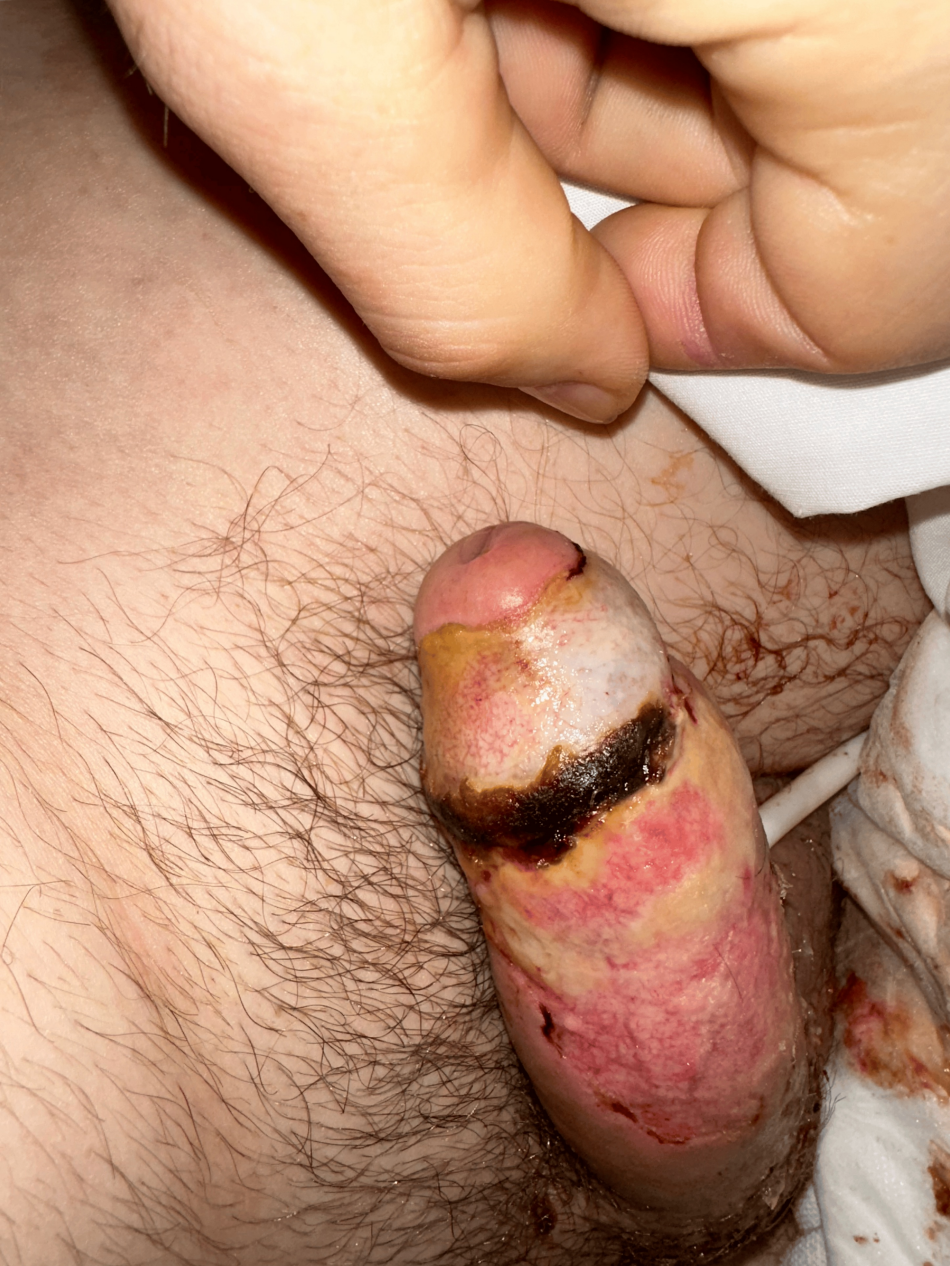
Periurethral sparing of the penile skin at re-admission.

The patient elected to discharge 48 hours after the initial consult. Two weeks later, the patient returned to the emergency department with worsening penoscrotal pain and recurrence of priapism. At this second dermatological examination, ischemic and necrotic skin of the penis had sloughed off, exposing the sharply demarcated distribution of skin necrosis, while other gross anatomical structures of the penis remained preserved (Figure 2). Due to persistent and recurring priapism, the patient subsequently underwent placement of a malleable penile prosthesis. Post-operation, the patient complained of persistent penile pain despite the use of patient-controlled analgesia. Ultimately, he was discharged three days later after transitioning to oral pain medication. He did not present to subsequent appointments and was lost to follow-up.

## Discussion

Our case presents a novel cutaneous manifestation of trazodone-induced priapism: vesiculobullous necrosis with periurethral sparing (Figure 2). This finding extends our understanding of the spectrum of complications that can arise from priapism and highlights the importance of recognizing its atypical presentations.

Penile necrosis is a very rare complication due to the organ’s rich collateral blood supply. There have been rare reports of ischemic priapism leading to gangrene of the penis, with underlying causes including sickle cell disease, thrombotic thrombocytopenic purpura, trauma, and multiple myeloma [3-5]. To our knowledge, priapism leading to penile bullous ischemic necrosis has never been reported.

The pathophysiological link between trazodone use and the vesiculobullous lesions observed in our patient likely involves multiple mechanisms. Trazodone’s alpha-adrenergic blockade properties directly contribute to priapism by interfering with normal detumescence mechanisms [2,6,7]. This pharmacological effect creates the sustained high intracavernosal pressures characteristic of ischemic priapism. The unusual distribution of vesiculobullous lesions with periurethral sparing observed in this case reflects the unique vascular architecture of the penis. The glans and periurethral tissues receive blood from the terminal branches of the internal pudendal artery via the dorsal penile artery and bulbourethral branches. This redundant blood supply creates a protective effect against severe ischemia in these regions. Moreover, the corpus spongiosum surrounding the urethra functions as a distinct compartment from the corpora cavernosa, typically experiencing lower pressures during ischemic priapism and thereby preserving periurethral blood flow.

This distinctive pattern of bullous ischemic necrosis can be attributed to a compartmental pressure phenomenon. During priapism, as intracavernosal pressures exceed mean arterial pressure, cutaneous microcirculation becomes compromised, particularly in watershed areas dependent on superficial vessels. Rather than frank gangrene, the vesiculobullous reaction suggests a partial-thickness ischemic injury sufficient to disrupt the dermal-epidermal junction but not severe enough to cause full-thickness necrosis. The 48-hour delay before lesion appearance aligns with ischemia-reperfusion injury, where restored blood flow after decompression triggers inflammatory cascades and oxidative damage to previously ischemic tissues.

While histopathological examination was not performed, we hypothesize that microscopic findings would have revealed subepidermal vesiculation with minimal inflammatory infiltrate, consistent with ischemia-induced dermal-epidermal separation rather than primary immunobullous disease. The papillary dermis would likely have shown endothelial swelling, microthrombosis, and erythrocyte extravasation, reflecting the suspected ischemia-reperfusion mechanism.

The differential diagnosis for this presentation is broad and includes other vascular-mediated conditions (Table 1). Bullous eruptions due to vascular insufficiency have been reported in other contexts, such as on the legs [8]. There are reports of bullae arising from chronic venous stasis, thought to be exceptional cases of localized bullous pemphigoid (BP) [9]. Localized BP is a specialized form that may appear on wound sites, surgical incisions, radiation-affected areas, or in venous stasis [10,11].

**Table 1:** Differential diagnosis for vesiculobullous penile lesions.

Differential diagnosis	Key features	Evidence for	Evidence against
Bullous ischemic necrosis	Sharp demarcation; distribution follows vascular territories; serous fluid	Timing after priapism; sparing of areas with dual blood supply	No precedent cases in the literature
Allergic contact dermatitis	Pruritic; erythematous base; vesiculation	Sharp demarcation; patient’s atopic history	Absence of pruritus; inexplicable distribution
Bullous fixed drug eruption	Recurs in the same location with drug re-exposure	Drug exposure (trazodone)	Atypical morphology; lack of violaceous hue; no prior history
Linear IgA bullous dermatosis	Triggered by medications; tense bullae	Bullous morphology	Limited distribution; abrupt onset
Necrotizing fasciitis	Rapidly progressive; systemic toxicity	Bullae formation	Absence of fever or systemic toxicity; limited distribution

Several underlying conditions that can lead to both priapism and cutaneous manifestations should be considered. IgA vasculitis predominantly affects the skin, joints, gastrointestinal tract, and kidneys, with rare penile involvement causing thrombosis and priapism [12,13]. While purpura and petechiae are the most common lesions, erythematous, bullous, macular, or urticarial features can also occur [14]. Similarly, protein C/S deficiencies may predispose to both skin necrosis and priapism through dysregulated coagulation, particularly during anticoagulant therapy [15-18]. These conditions highlight the interconnection between vascular, coagulative, and inflammatory pathways in the pathogenesis of both priapism and cutaneous necrosis.

It is possible that skin necrosis developed due to localized pressure from the surgical intervention, but the marked sparing of the periurethral glans and sharply demarcated vesiculation at the base of the penis are difficult to explain by pressure-related ischemia alone. This is particularly notable given the rich arterial supply to the penile skin from the internal and external pudendal arteries [19,20]. Penoscrotal decompression procedure has not been reported in association with the bullous ischemic necrosis observed in this case.

This case has several limitations. First, the patient was lost to follow-up, limiting assessment of long-term outcomes such as resolution, recurrence, or complications. Additionally, no biopsy was performed, which limits diagnostic certainty. Although the clinical presentation strongly suggests bullous ischemic necrosis, histopathologic confirmation would have strengthened this diagnosis. Future cases should consider biopsy when feasible to help distinguish ischemic injury from other potential etiologies.

## Conclusions

Ultimately, this case has significant clinical implications. First, it demonstrates that vesiculobullous necrosis with periurethral sparing can occur as a delayed complication of trazodone-induced priapism. Second, it highlights the importance of understanding penile vascular anatomy when evaluating cutaneous manifestations in this region. Third, it reinforces the need for vigilant monitoring of skin integrity following priapism procedures. Recognition of the distinctive pattern of periurethral sparing may help differentiate vascular compromise from primary cutaneous disorders, guiding appropriate management decisions in similar cases.
